# Supplementary material for: PANDA: A comprehensive and flexible tool for quantitative proteomics data analysis
Source: Bioinformatics. 2018 Aug 23;35(5):898–900. doi: 10.1093/bioinformatics/bty727 (PMC6394390; doi:10.1093/bioinformatics/bty727)
Supplement: Supplementary Data [file bty727_supp.zip › bty727-suppl_data/bty727_Supplementary_Information.docx]

PANDA: A comprehensive and flexible tool for proteomics data quantitative analysis

Cheng Chang^1,*,#^, Mansheng Li^1,#^, Chaoping Guo^2^, Yuqing Ding^2^, Kaikun Xu^1^, Mingfei Han^1^, Fuchu He^1^ and Yunping Zhu^1,*^

*^1^State key Laboratory of Proteomics, Beijing Proteome Research Center, Beijing Institute of Lifeomics, National Center for Protein Sciences (Beijing), Beijing 102206, P.R. China.*

*^2^Beijing Key Laboratory of Human Computer Interactions, Institute of Software, Chinese Academy of Sciences, Beijing 100190, P.R. China.*

^#^These authors contributed equally to this work.

^*^Correspondence should be addressed to:

Cheng Chang (1987ccpacer@163.com)

Yunping Zhu (zhuyunping@gmail.com)

# Supplementary Methods

## Experimental datasets

**The yeast dataset.** To evaluate the accuracy and precision of label-free quantification, the yeast mass spectrometry (MS) data obtained from ([Chang, et al., 2016](#_ENREF_2)) was used in this study. In this dataset, a series of UPS2 standard proteins (Proteomics Dynamic Range Standard, Sigma-Aldrich) with four levels of amounts (1µg, 0.2µg, 0.04µg, 0.008µg) were spiked into the yeast samples, named as A-D groups. The loading amount of yeast samples were equal in all the four groups. Each group contains three technique replicates. The UPS2 standard proteins consist of 48 synthesized proteins with six levels of concentrations, ranging from 5000 fmol to 0.05 fmol. The MS data is public available in the ProteomeXchange Consortium via the iProX partner repository (http://www.iprox.org) with the identifier PXD008428.

**The HeLa dataset.** This dataset was obtained by analyzing the MS data from ([Cox and Mann, 2008](#_ENREF_3)) and used for evaluation of labeled quantification. The unlabeled HeLa cells were mixed with the same amount of SILAC labeled samples. The SILAC labels consist of lysine with +8.014199Da and arginine with +10.008269Da. The mixed samples were separated by isoelectric focusing into 24 fractions and analyzed by MS in triplicate. The MS data was available in Tranche at http://proteomecommons.org/tranche as described in the corresponding paper.

**The phosphorylation datasets with SILAC and TMT labeling methods**. The raw MS data downloaded from ([Hogrebe, et al., 2018](#_ENREF_6)) were re-analyzed and used to evaluate quantification accuracy and precision for large-scale phosphoproteomics. In this dataset, diluted phosphopeptides enriched from yeast at fixed 1:4:10 ratios into a 1:1:1 background of HeLa phosphopeptides. The dilution series are named as A, B and C in this study. The SILAC 3-plex (Lys 0, Lys 4 and Lys 8) and TMT 10-plex (1-I; 4-I; 10-I; 1-II; 4-II; 10-II; 1-III; 4-III; 10-III; pool) labeling methods were used for the diluted phosphopeptides. The SILAC phosphorylation datasets have two groups (i.e., the high injection load group and the low injection load group). For the TMT phosphorylation datasets, only the MS2-based TMT labeling data were used in this study, which also have two groups (i.e., the high injection load and the low injection load). The high injection group has two technical replicates and the low injection group has three technical replicates. All samples were measured on an Orbitrap Fusion Lumos. The detailed information about these phosphorylation datasets can be found in Supplementary Table 2. The data is available in the ProteomeXchange Consortium via the PRIDE partner repository (https://www.ebi.ac.uk/pride/archive/) with the identifier PXD007145.

**The Arabidopsis dataset** The MS data with different ^15^N labeling ratios (^15^N: ^14^N is 1:2, 1:5 and 1:10) were downloaded from ([Arsova, et al., 2012](#_ENREF_1)) for evaluation of quantification accuracy. The MS data was available in Tranche (http://proteomecommons.org/tranche) as described in the corresponding paper.

## Peptide identification

For PANDA, all MS data from the datasets mentioned above were re-analyzed as follows: MS raw files were processed by msconvert (ProteoWizard suite version 3.0.11516) using the default parameters. The acquired MS/MS peak list files (MGF files) were searched by Mascot (version 2.3.2) search engine against the Swiss-Prot yeast database (release 2013_11) with 48 UPS2 standard proteins sequences for the yeast dataset, the Swiss-Prot human database (release 2013_06) for the HeLa dataset and the yeast-human mixed database for the phosphorylation datasets. The detailed search parameters were kept the same as the descriptions in their original papers. The Mascot results were processed using PepDistiller ([Li, et al., 2012](#_ENREF_9)) for quality control of the peptide identifications.

For MaxQuant, all MS raw files were loaded into MaxQuant (v1.6.0.13) for peptide identification and quantification. MS data were searched by Andromeda ([Cox, et al., 2011](#_ENREF_4)) against the same protein sequence databases mentioned above. The search parameters were also kept unchanged for a fair comparison of PANDA and MaxQuant.

For quality control of the peptide identification results, both peptide and protein false discovery rates (FDRs) were kept below 0.01 in this study.

Especially, for the ^15^N labeling data, all MS data are searched against the Arabidopsis protein database (TAIR10, release 20101214) using Mascot (v2.3.2) with the following parameters. (1) Precursor and product ion mass tolerances with 20 ppm and 1.0 Da, respectively; (2) Trypsin enzyme; (3) A maximum of two missed cleavages; (4) Carbamidomethylation cysteine (+57.0215 Da) as a fixed modification; (5) Oxidation (+15.9949 Da) on methionine as a variable modification; (6) ^15^N Metabolic [MD] for searching ^15^N labeling data. The acquired Mascot result files are processed in Trans-Proteomic Pipeline ([Deutsch, et al., 2010](#_ENREF_5)) using PeptideProphet ([Keller, et al., 2002](#_ENREF_8)) for quality control. Then, the resulted peptide identifications are used as the input of PANDA for peptide and protein quantification by choosing the precursor labeling method as ^15^N.

# Supplementary Notes

## 1 Quantification algorithms in PANDA

At spectrum level, PANDA performs isotopic peak detection using the user-defined mass tolerance. Then the extracted ion current (XIC) is constructed for every peptide-spectrum match (PSM) between the user-defined retention time (RT) range. The signal-to-noise (S/N) ratio and the goodness of least-square fitting between the theoretical and observed isotope distributions are calculated as two cutoffs to filter out the unconfident PSM quantification results. The integral area of each XIC is calculated as the PSM quantification result. (2) At peptide level, the peptide intensity is defined as the average of the corresponding XIC areas in multiple PSMs. (3) At protein level, PANDA first performs protein inference based on the parsimony principle, i.e., Ockham’s razor ([Huang, et al., 2012](#_ENREF_7); [Yang, et al., 2004](#_ENREF_10)), which is a mainstream principle for protein inference and also used in MaxQuant. Second, in PANDA, only the protein groups with at least one unique peptide are retained for protein quantification. The intensity of a protein group is defined as the summed intensities of its corresponding unique peptides. To make a fair comparison, in this study, we also only choose unique peptides for protein quantification in MaxQuant.

## 2 Quantification accuracy evaluation

To evaluate the quantification accuracy of PANDA and MaxQuant, the commonly quantified proteins by the two software tools are selected for further comparison. Before the selection, the technical replicates are merged at first in either dataset as follows:

$$protein merged intensity=\frac{\sum_{i=1}^{N} {protein intensity}_{i}}{N}$$

where N is the number of replicates with intensity larger than zero, ${protein intensity}_{i}$ is the protein intensity in the *i-th* replicate.

In the yeast dataset, there were 20 UPS2 proteins quantified in all the four groups (A-D) by PANDA and MaxQuant. The theoretical ratios of these proteins for A/B, A/C and A/D are 5, 25, 125. As shown in Supplementary Figure 1a, PANDA showed a closer ratio distribution to the theoretical value than MaxQuant in all the situations. Moreover, when we split these proteins with their actual amounts, the results showed the similar trends (Supplementary Figure 1b-d). Note that the 20 UPS2 proteins consist of all the 16 proteins in 5000 and 500 fmol levels, as well as four proteins in 50 fmol level. Due to the very few number of quantified proteins in 50 fmol level, the results from 50 fmol level are easily influenced by outliers and thus not so reliable. Therefore, only the proteins in 5000 fmol and 500 fmol levels were shown in boxplot in Supplementary Figure 1b-d.

In the HeLa dataset, after merging the three technical replicates, there were 3471 proteins both quantified by PANDA and MaxQuant. As shown in Supplementary Figure 2, the protein SILAC ratios of PANDA were significantly closer to the theoretical value (1:1) than those of MaxQuant with Wilcoxon rank sum test *p-value*<0.001. The median ratio of these proteins quantified by PANDA is 0.85, while the median ratio of MaxQuant is 0.78.

In the phosphorylation SILAC 3-plex labeling datasets, there were 164 and 131 yeast phosphopeptides both quantified by PANDA and MaxQuant in all the dilution series (1:4:10) for the high and low injection groups, respectively and there were 4521 and 4099 human phosphopeptides both quantified by PANDA and MaxQuant in all the dilution series (1:1:1) for the high and low injection groups, respectively. The phosphopeptide ratios in the dilution series are shown in Supplementary Figure 3. For yeast and human peptides, PANDA has a similar or an even better accuracy compared with MaxQuant in both high and low injection groups.

In the phosphorylation TMT 10-plex labeling datasets, there were 1152 and 1020 yeast phosphopeptides both quantified by PANDA and MaxQuant in all the dilution series (1:4:10) for the high and low injection groups, respectively. And there were 4196 and 3936 human phosphopeptides both quantified by PANDA and MaxQuant in all the dilution series (1:1:1) for the high and low injection groups, respectively. As shown in Supplementary Figure 4, the same trend was observed. All these results demonstrate that PANDA could accurately quantify large-scale phosphoproteomic data.

In the ^15^N labeling dataset, the protein ratios (^15^N: ^14^N) were calculated and shown in Supplementary Figure 5. We can find that the median ratios of the quantified proteins in the three samples were all close to their theoretical values (1:2, 1:5 and 1:10), demonstrating that PANDA can handle ^15^N labeling data with high accuracy.

To sum up, we can conclude that PANDA owns a high accuracy for label-free and labeled quantifications in a wide dynamic range.

## 3 Quantification precision evaluation

In the yeast dataset, since the loading amount of the yeast samples remained the same in A-D groups as background, the coefficient of variations (CVs) of the three technical replicates within each group were calculated respectively to evaluate the precision of the technical replicates for label-free quantification. As shown in Supplementary Figure 6, there was an obvious difference between the protein CV distributions within each group using PANDA and MaxQuant indicating that PANDA is of high precision for label-free quantification.

In the HeLa dataset, there were 1905 and 1958 proteins quantified in all the three technical replicates with two or more spectral counts using PANDA and MaxQuant, respectively. Among them, a total of 1573 proteins were both quantified by the two software tools. As shown in Supplementary Figure 7, the CV distribution of PANDA is significantly closer to zero than that of MaxQuant for both heavy and light labeling samples (Wilcoxon rank sum test *p-value*<0.001), proving that PANDA is also precise for labeled quantification.

## 4 Summary of the features in PANDA

PANDA has the following features in terms of functionality and performance: (1) Fully support the HUPO/PSI standard data formats (mzXML, mzML and mzIdentML). (2) Support label-free quantification, i.e., spectral count (SC) or intensity-based quantification. (3) Support a variety of labeling methods (such as SILAC, ^18^O, ICAT, ICPL, iTRAQ and TMT), especially the ^15^N labeling method. While, MaxQuant and Proteome Discoverer cannot deal with ^15^N labeling data. (4) Allow users to define new labeling methods according to their data. (5) Accurate and efficient. However, PANDA can only run in Windows at present. Other operating systems will be supported in the future.

# Supplementary References

Arsova, B., Zauber, H. and Schulze, W.X. (2012) Precision, proteome coverage, and dynamic range of Arabidopsis proteome profiling using (15)N metabolic labeling and label-free approaches, *Mol Cell Proteomics*, **11**, 619-628.

Chang, C.*, et al.* (2016) Quantitative and In-Depth Survey of the Isotopic Abundance Distribution Errors in Shotgun Proteomics, *Anal Chem*, **88**, 6844-6851.

Cox, J. and Mann, M. (2008) MaxQuant enables high peptide identification rates, individualized p.p.b.-range mass accuracies and proteome-wide protein quantification, *Nat Biotechnol*, **26**, 1367-1372.

Cox, J.*, et al.* (2011) Andromeda: a peptide search engine integrated into the MaxQuant environment, *J Proteome Res*, **10**, 1794-1805.

Deutsch, E.W.*, et al.* (2010) A guided tour of the Trans-Proteomic Pipeline, *Proteomics*, **10**, 1150-1159.

Hogrebe, A.*, et al.* (2018) Benchmarking common quantification strategies for large-scale phosphoproteomics, *Nature communications*, **9**, 1045.

Huang, T.*, et al.* (2012) Protein inference: a review, *Brief Bioinform*, **13**, 586-614.

Keller, A.*, et al.* (2002) Empirical statistical model to estimate the accuracy of peptide identifications made by MS/MS and database search, *Anal Chem*, **74**, 5383-5392.

Li, N.*, et al.* (2012) PepDistiller: A quality control tool to improve the sensitivity and accuracy of peptide identifications in shotgun proteomics, *Proteomics*, **12**, 1720-1725.

Yang, X.*, et al.* (2004) DBParser: web-based software for shotgun proteomic data analyses, *J Proteome Res*, **3**, 1002-1008.

# Supplementary Figures and Tables


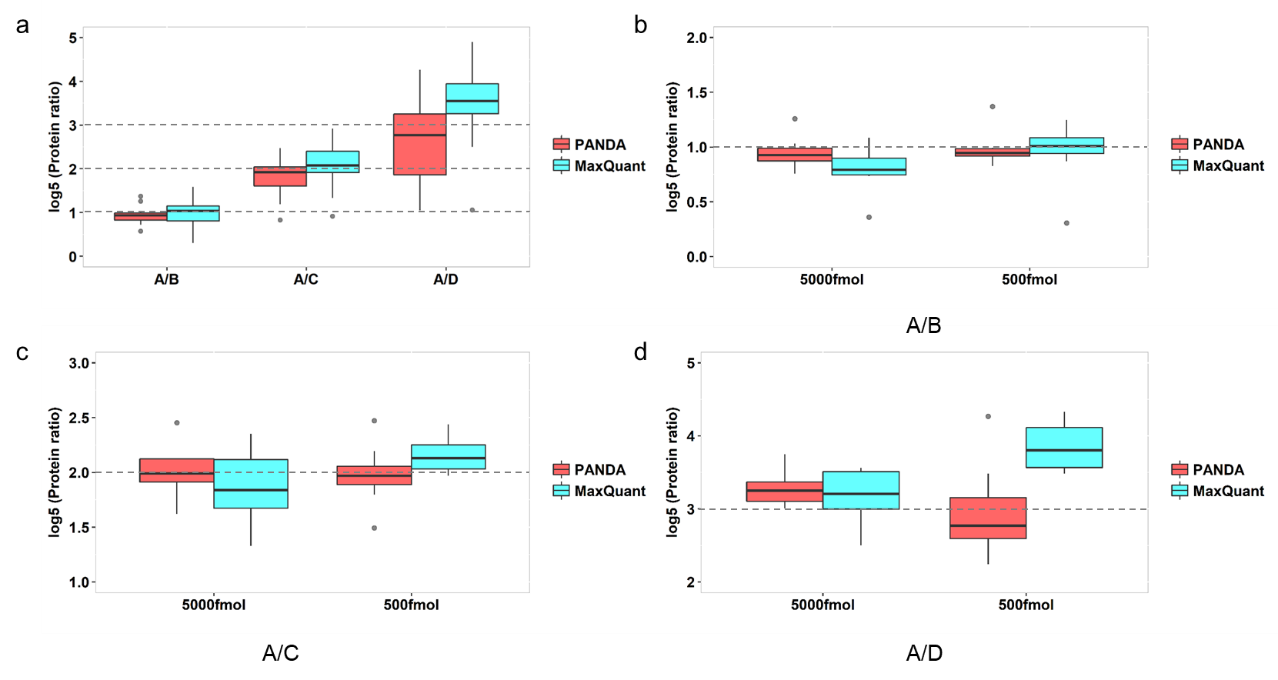


**Supplementary Figure 1.** Accuracy evaluation for label-free quantification. Boxplots of the spike-in UPS2 protein ratios between A-D groups in the yeast dataset using PANDA (red) and MaxQuant (cyan) separately. A-D indicate the four dilution concentrations of the UPS2 proteins spiked in the yeast samples. (a) Protein ratio boxplots of all the UPS2 proteins for A/B, A/C and A/D. (b-d) Protein ratio boxplots of UPS2 proteins with 5000 fmol and 500 fmol, respectively. The protein ratios are shown in base-5 logarithm scale. The gray dashed lines represent the theoretical ratios. In all boxplots, the center black line is the median of the protein quantification errors; the box limits are the upper and lower quartiles; the whiskers delimit the most extreme data points within 1.5 interquartile range below the first quartile and above the third quartile, respectively.


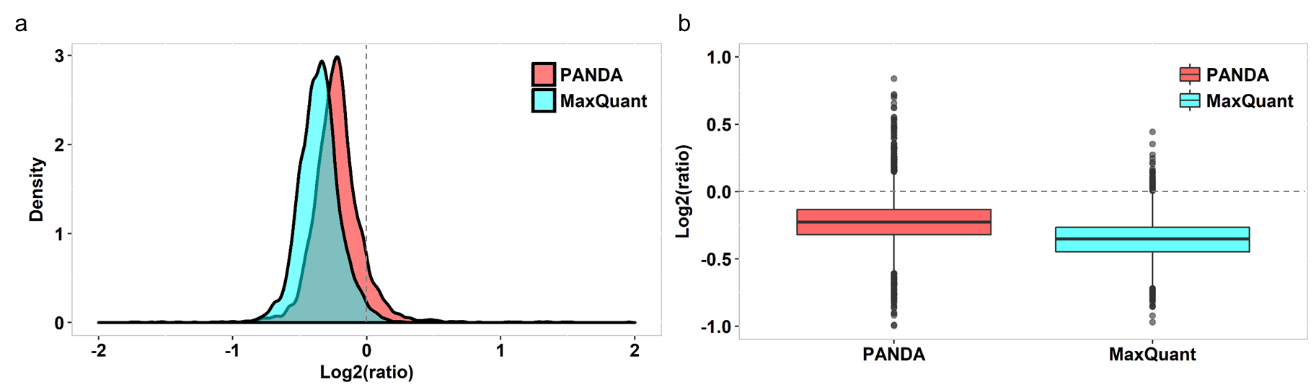


**Supplementary Figure 2.** Accuracy evaluation for labeled quantification. Distributions of the protein ratios between the SILAC labeled and unlabeled samples in the HeLa dataset using PANDA (red) and MaxQuant (cyan). (a) Density plots of the protein ratios commonly quantified by PANDA and MaxQuant. (b) Boxplots of the protein ratios commonly quantified by PANDA and MaxQuant. The protein ratios are shown in base-2 logarithm scale. The gray dashed lines represent the theoretical ratios. In the boxplot, the center black line is the median of the protein quantification errors; the box limits are the upper and lower quartiles; the whiskers delimit the most extreme data points within 1.5 interquartile range below the first quartile and above the third quartile, respectively.


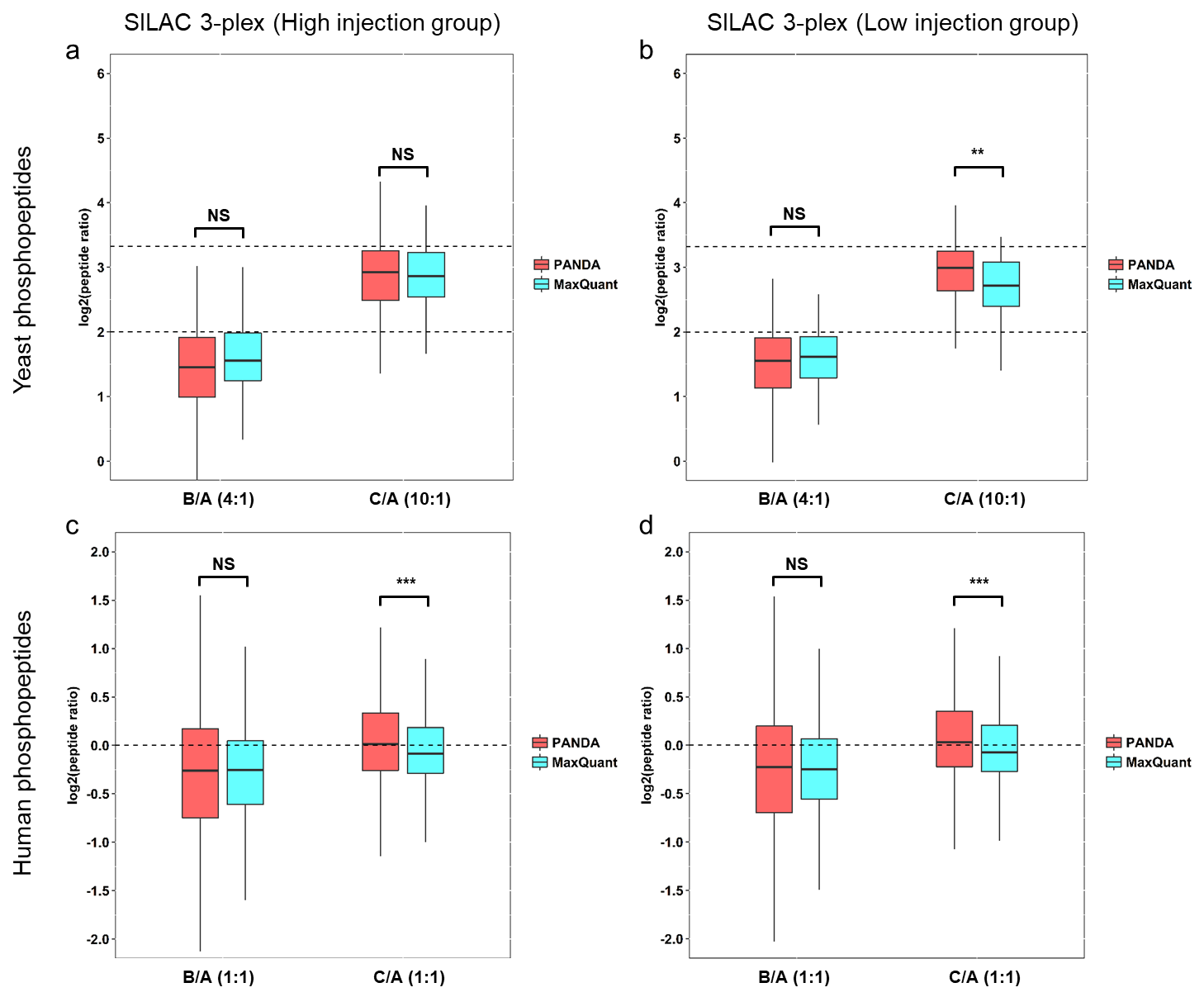


**Supplementary Figure 3.** Accuracy evaluation for phosphoproteomic data with SILAC 3-plex labeling method. Distributions of the phosphopeptide ratios in the dilution series A/B/C (1:4:10 for yeast samples and 1:1:1 for HeLa samples) calculated by PANDA (red) and MaxQuant (cyan) are shown in boxplots. (a) and (c) are from the high injection group; (b) and (d) are from the low injection group. The peptide ratios are shown in base-2 logarithm scale. The gray dashed lines represent the theoretical ratios. In all boxplots, the center black line is the median of the protein quantification errors; the box limits are the upper and lower quartiles; the whiskers delimit the most extreme data points within 1.5 interquartile range below the first quartile and above the third quartile, respectively. ^***^P<0.001, ^**^P<0.01, ^*^P<0.05, NS means not significant (Wilcoxon rank-sum test).


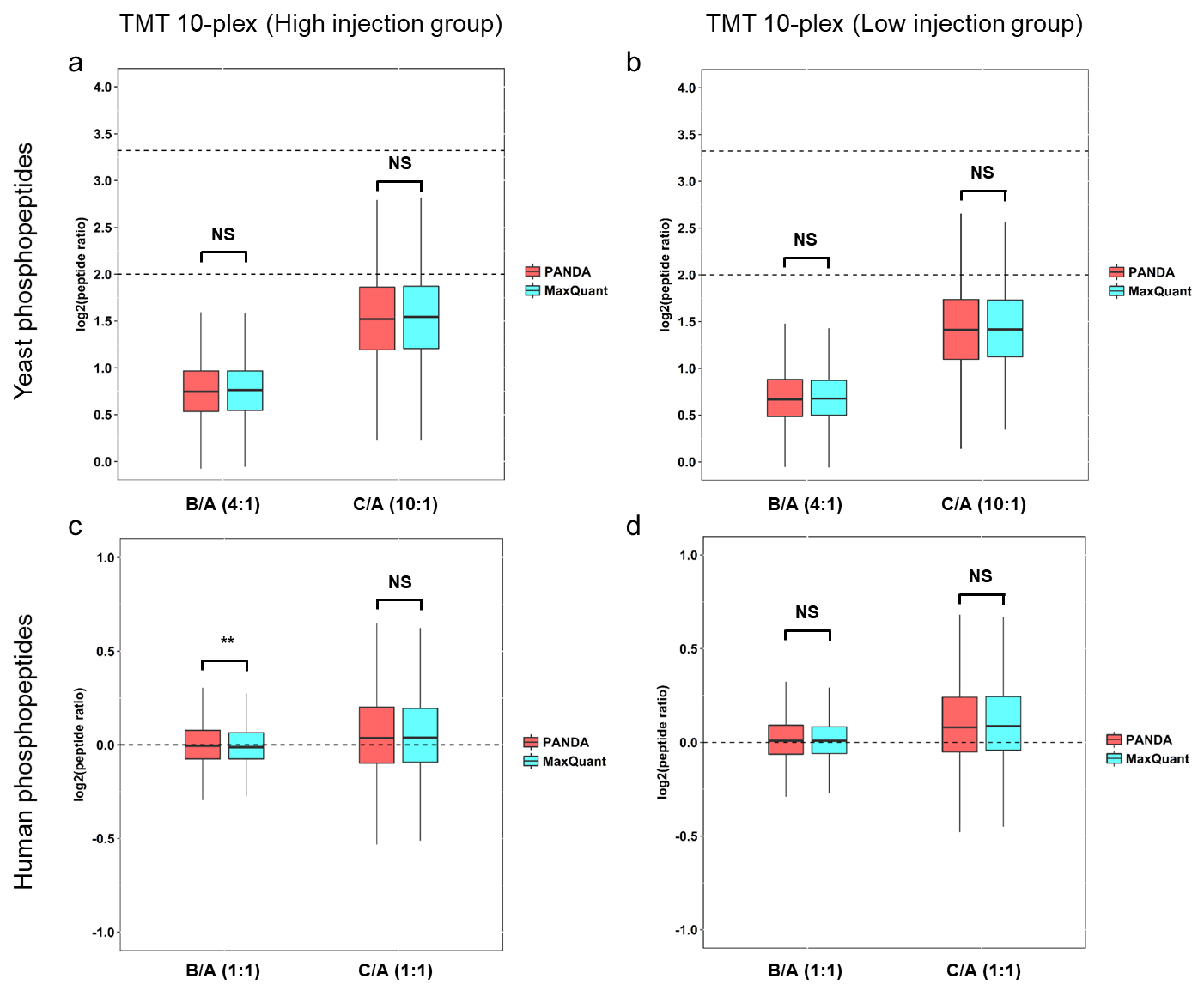


**Supplementary Figure 4.** Accuracy evaluation for phosphoproteomic data with TMT 10-plex labeling method. Distributions of the phosphopeptide ratios in the dilution series A/B/C (1:4:10 for yeast samples and 1:1:1 for HeLa samples) calculated by PANDA (red) and MaxQuant (cyan) are shown in boxplots. (a) and (c) are from the high injection group; (b) and (d) are from the low injection group. The peptide ratios are shown in base-2 logarithm scale. The gray dashed lines represent the theoretical ratios. In all boxplots, the center black line is the median of the protein quantification errors; the box limits are the upper and lower quartiles; the whiskers delimit the most extreme data points within 1.5 interquartile range below the first quartile and above the third quartile, respectively. ^***^P<0.001, ^**^P<0.01, ^*^P<0.05, NS means not significant (Wilcoxon rank-sum test).


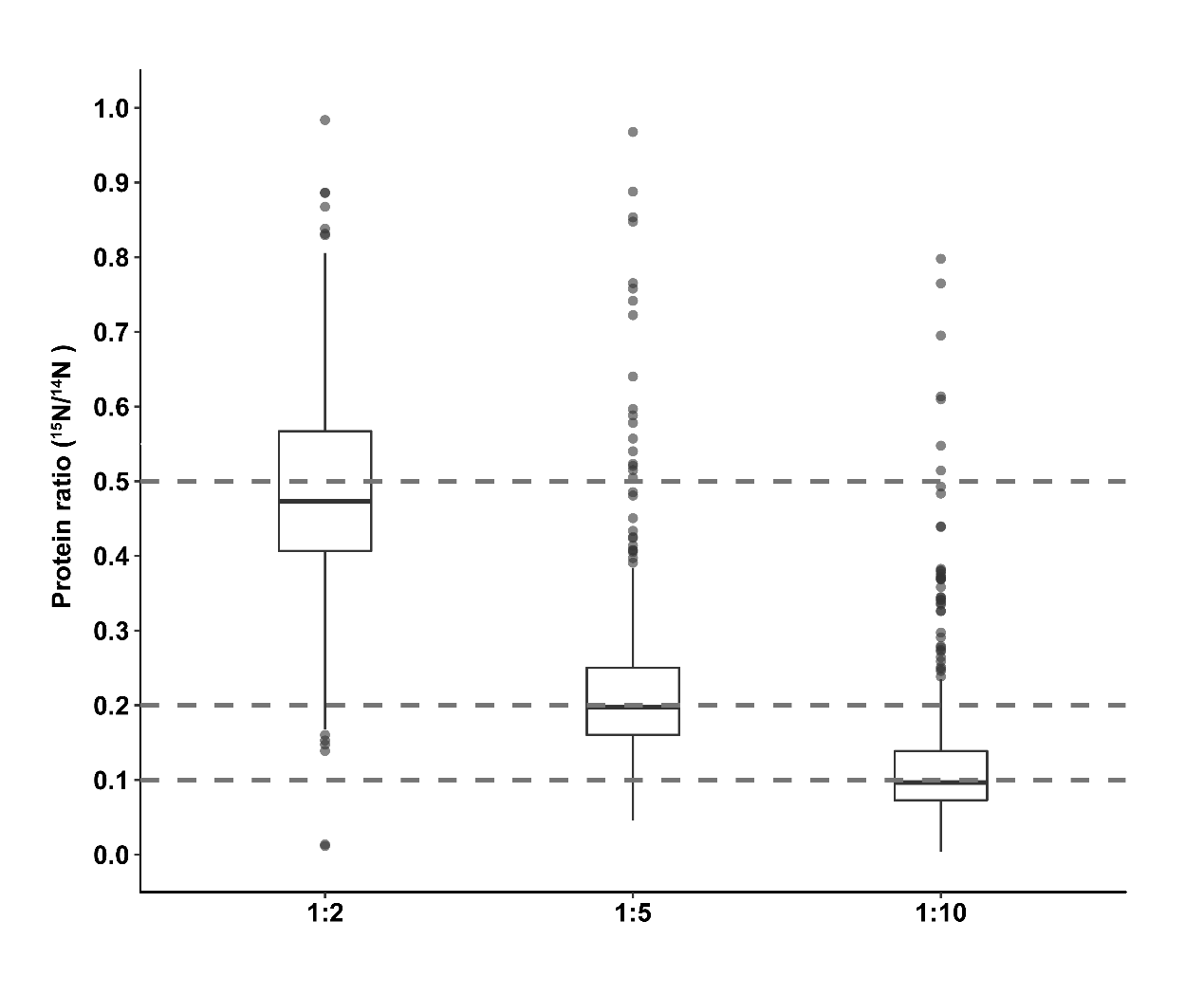


**Supplementary Figure 5.** Quantification accuracy evaluation of PANDA on a public ^15^N labeling dataset with three different labeling ratios (^15^N/^14^N=1:2, 1:5 and 1:10). The dashed gray lines represent the theoretical ratios. In all boxplots, the center black line is the median of the protein quantification errors; the box limits are the upper and lower quartiles; the whiskers delimit the most extreme data points within 1.5 interquartile range below the first quartile and above the third quartile, respectively.


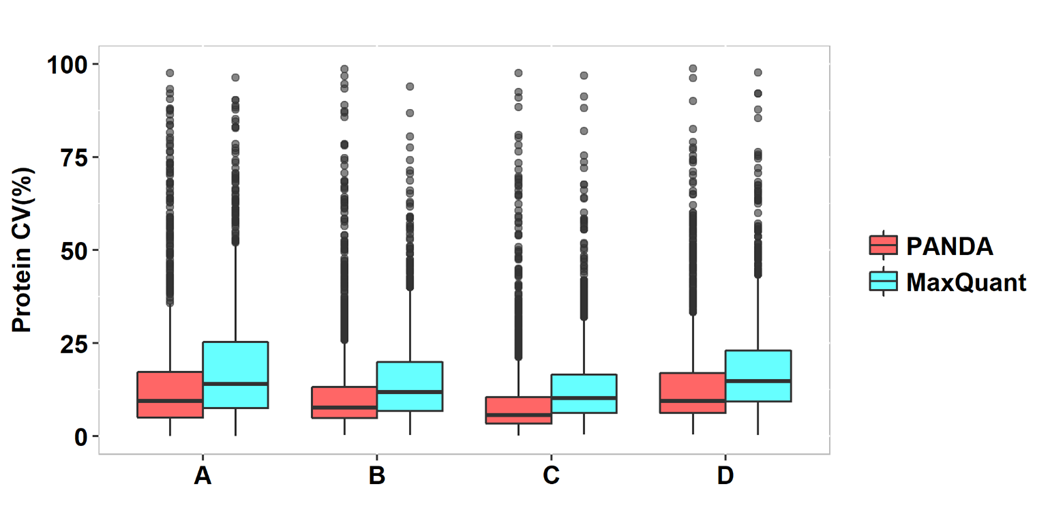


**Supplementary Figure 6.** Precision evaluation for label-free quantification on the yeast dataset. Boxplots of yeast protein intensity CVs of the three technical replicates within each group (A-D). The red box indicates PANDA and the cyan one indicates MaxQuant. In all boxplots, the center black line is the median of the protein quantification errors; the box limits are the upper and lower quartiles; the whiskers delimit the most extreme data points within 1.5 interquartile range below the first quartile and above the third quartile, respectively.

**
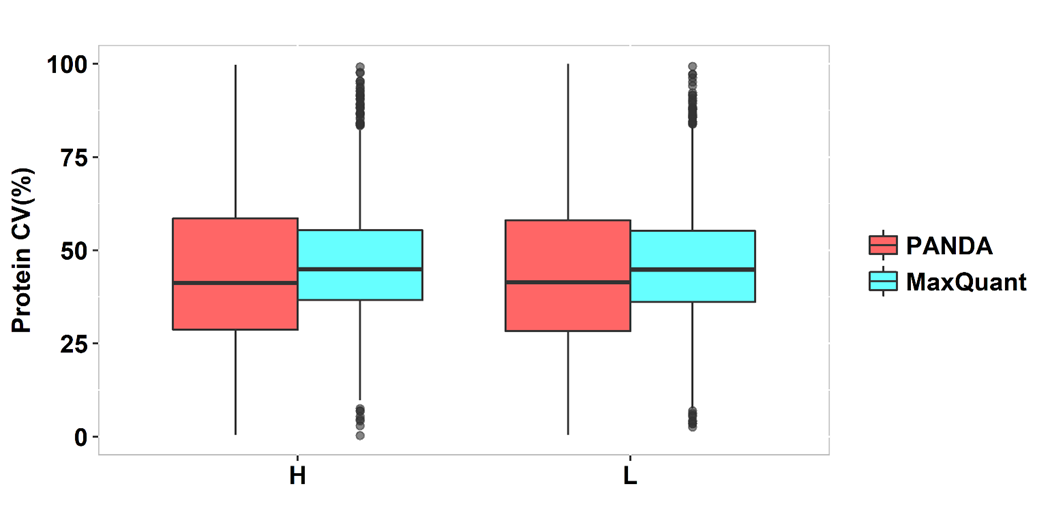
**

**Supplementary Figure 7.** Precision evaluation for labeled quantification on the HeLa dataset. Boxplots of the human protein intensity CVs of the three replicates in the HeLa dataset. H and L indicate SILAC labeled (heavy) and unlabeled samples (light), respectively. In all boxplots, the center black line is the median of the protein quantification errors; the box limits are the upper and lower quartiles; the whiskers delimit the most extreme data points within 1.5 interquartile range below the first quartile and above the third quartile, respectively.

**Supplementary Table 1.** List of the quantification time (in minute) of PANDA and MaxQuant for the datasets used in this study.

| Dataset | Reference | MaxQuant v1.6.0.13 | PANDA v1.1.3 |
| --- | --- | --- | --- |
| Yeast | 1 | 35 | 16 |
| HeLa | 2 | 133 | 27 |
| SILAC high injection group | 3 | 31 | 6.2 |
| SILAC low injection group | 3 | 28 | 5.5 |
| TMT high injection group | 3 | 16 | 3.2 |
| TMT low injection group | 3 | 21 | 4.3 |
| Arabidopsis (^15^N labeling) | 4 | Not available | 5.0 |

**Note:**

1. For MaxQuant, only the quantification time (i.e. starting from the step "Re-quantification" to the last step) was considered, not including the time for data searching and quality control.
2. PANDA and MaxQuant were tested using one thread on the same computer: Windows7 64-bit operating system, Intel Core E3–1230 v3 CPU 3.30-GHz processors, 2 TB SATA3 hard disk with 7200 rpm, and 8 GB RAM.

**References:**

1. Chang, C., et al. (2016) Quantitative and In-Depth Survey of the Isotopic Abundance Distribution Errors in Shotgun Proteomics, Anal Chem, 88, 6844-6851.
2. Cox, J. and Mann, M. (2008) MaxQuant enables high peptide identification rates, individualized p.p.b.-range mass accuracies and proteome-wide protein quantification, Nat Biotechnol, 26, 1367-1372.
3. Hogrebe, A., et al. (2018) Benchmarking common quantification strategies for large-scale phosphoproteomics, Nature communications, 9, 1045.
4. Arsova, B., Zauber, H. and Schulze, W.X. (2012) Precision, proteome coverage, and dynamic range of Arabidopsis proteome profiling using (15)N metabolic labeling and label-free approaches, Mol Cell Proteomics, 11, 619-628.

**Supplementary Table 2.** Detailed descriptions of the MS data from Hogrebe, A., et al.’s paper which were used in this study. Supplementary Table 2 is provided as a separate Excel file.
