# Supplementary material for: PANDA: A comprehensive and flexible tool for quantitative proteomics data analysis
Source: Bioinformatics. 2018 Aug 23;35(5):898–900. doi: 10.1093/bioinformatics/bty727 (PMC6394390; doi:10.1093/bioinformatics/bty727)
Supplement: Supplementary Data [file bty727_supp.zip › bty727-suppl_data/bty727_Supplementary_Manual.docx]

| 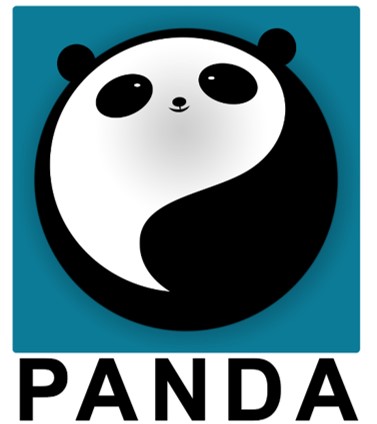  A COMPREHENSIVE AND FLEXIBLE TOOL FOR PROTEOMICS DATA QUANTITATIVE ANALYSIS | **User Manual for PANDA**  Author: Dr. Cheng Chang  Address: State Key Laboratory of Proteomics, Beijing Proteome Research Center, Beijing 102206, China |
| --- | --- |

**Contents**

[Chapter 1. General Information 1](#_Toc502227840)

[1.1 Software Overview 1](#_Toc502227841)

[1.2 Organization of the Manual 1](#_Toc502227842)

[Chapter 2. Getting Started 3](#_Toc502227843)

[2.1 Installation 3](#_Toc502227844)

[2.1.1 Requirement 3](#_Toc502227845)

[2.1.2 Download 3](#_Toc502227846)

[2.2 Menu 4](#_Toc502227847)

[2.2.1 Language 4](#_Toc502227848)

[2.2.2 Help 5](#_Toc502227849)

[2.2.3 About 5](#_Toc502227850)

[2.2.4 Exit 6](#_Toc502227851)

[Chapter 3. Main Interface 7](#_Toc502227852)

[3.1 Parameter setting in the *Data* interface 7](#_Toc502227853)

[3.1.1 Configuration file 7](#_Toc502227854)

[3.1.2 Input data type 9](#_Toc502227855)

[3.1.3 Experiment design setting 9](#_Toc502227856)

[3.1.4 Loading MS data and peptide identifications 11](#_Toc502227857)

[3.2 Parameter setting in the *Parameter* interface 11](#_Toc502227858)

[3.2.1 Label-free quantification 11](#_Toc502227859)

[3.2.2 Labeled quantification 12](#_Toc502227860)

[3.3 Parameter setting in the *Progress* interface 14](#_Toc502227861)

[Chapter 4. Output Files 17](#_Toc502227862)

[Chapter 5. Support Services 18](#_Toc502227863)

[5.1 Contact 18](#_Toc502227864)

[5.2 Copyright 18](#_Toc502227865)

[Chapter 6. References 19](#_Toc502227866)

# Chapter 1. General Information

## 1.1 Software Overview

PANDA is a comprehensive and flexible tool for quantitative proteomics data analysis, which is developed based on the progresses in quantitative proteomics of our lab for years ([Chang, et al., 2014](#_ENREF_2); [Chang, et al., 2016](#_ENREF_3); [Wang, et al., 2017](#_ENREF_10); [Zhang, et al., 2012](#_ENREF_11)). The advantage algorithms of LFQuant ([Zhang, et al., 2012](#_ENREF_11)) and SILVER ([Chang, et al., 2014](#_ENREF_2)) were implemented into PANDA including the reversible retention time (RT) alignment algorithm and the state-of-art concept of quantification reliability. PANDA can deal with both label-free and labeled quantifications and is very flexible to access the public standard data format such as mzXML ([Pedrioli, et al., 2004](#_ENREF_9)), mzML ([Deutsch, 2008](#_ENREF_4)) and mzIdentML ([Jones, et al., 2012](#_ENREF_6)). The core algorithm of PANDA was written in standard C++ language on the platform of Microsoft Visual Studio ultimate 2017 in Windows System. And the interfaces of PANDA were implemented in C# on the same platform.

When PANDA finishes quantification analysis, the quantification results of PANDA can be further analyzed in its affiliated tool PANDA-view ([Chang, et al., 2018](#_ENREF_1)) for statistical analysis and data visualization.

## 1.2 Organization of the Manual

The user’s manual consists of six chapters: General Information, Getting Started, Parameter Setting, Output Files, References and Supporting Services.

1. General Information chapter shows background information of PANDA.
2. Getting Started chapter explains how to download and install PANDA on the user’s computer.
3. Main Interface chapter provides a detailed description of Data, Parameters and Progress.
4. Output Files chapter explains the explains the features of PANDA quantification results.
5. References chapter lists the cited references in the manual.
6. Support Services chapter provides the author's contact information for consultation as well as the Copyright.

# Chapter 2. Getting Started

## 2.1 Installation

### 2.1.1 Requirement

1. Hardware requirements
2. Intel Pentium III/800 MHz or higher (or compatible) although one should probably not go below a dual core processor.
3. 2 GB RAM minimum.
4. Software requirements
   1. Supported OS versions (**64 bit is required**)

Windows 7 SP1

Windows Server 2008 R2 SP1

Windows Server 2008 SP2

Windows 8

Windows 10

- 1. [.NET Framework 4.5](https://www.microsoft.com/en-us/download/details.aspx?id=30653) or higher from Microsoft.
  2. MSFileReader: Both 32bit and 64bit versions are required to be installed to access Thermo raw files. The latest version MSFileReader 3.0 SP3 can be downloaded from [here](https://thermo.flexnetoperations.com/control/thmo/download?element=6306677).
  3. Microsoft Visual C++ Redistributable for Visual Studio 2017 (x64) can be downloaded from the [download link](https://go.microsoft.com/fwlink/?LinkId=746572).

### 2.1.2 Download

1. PANDA can be freely downloaded from <https://sourceforge.net/projects/panda-tools/>.
2. Un-compress the zip package (or 7z) into a specified file folder.
3. Double-click “PANDA.exe” and the graphical user interface (GUI) of PANDA will be shown (Fig. 1). Once the parameters were set in GUI or a configuration file was loaded, PANDA is ready to perform quantification analysis.


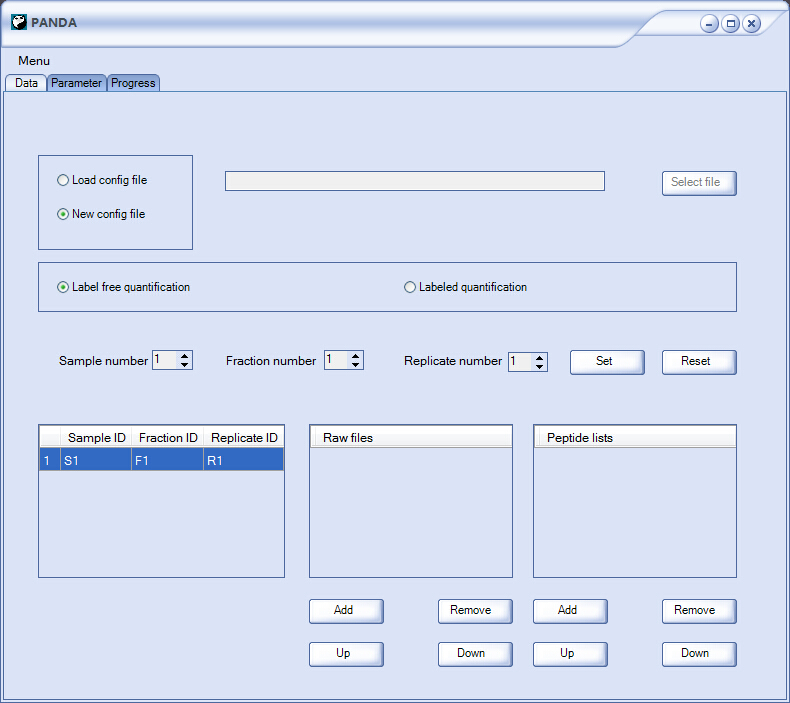


Figure 1. The GUI of PANDA for setting parameters.

## 2.2 Menu

### 2.2.1 Language

PANDA supports bilingual display. The software interface will switch between Simplified Chinese and English if users click the *Language* item in *Menu* (Fig. 2).


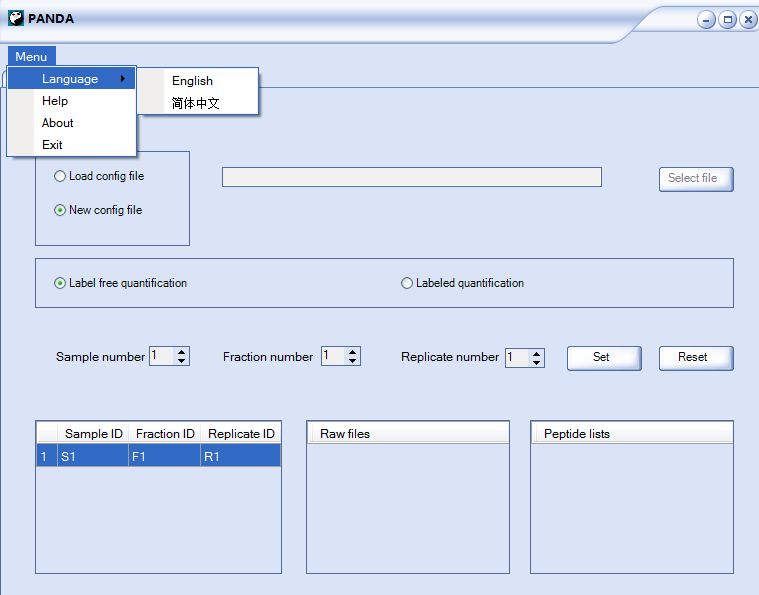


Figure 2. The *Language* item in *Menu*.

### 2.2.2 Help

Clicking the *Help* item in *Menu*, users can be directed to the PANDA website at SourceForge: <https://sourceforge.net/projects/panda-tools/>.

### 2.2.3 About

Some detailed information about the developers will be presented to users once the *About* item is clicked. (Fig. 3).


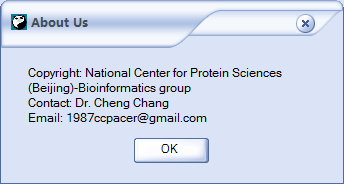


Figure 3. The *About* item in *Menu*.

### 2.2.4 Exit

To close PANDA, users can click the *Exit* item in *Menu* or click the close symbol at the top right corner of the GUI and a dialog will be shown (Fig. 4). Click *OK* to close PANDA, or click *Cancel* to stay in PANDA.


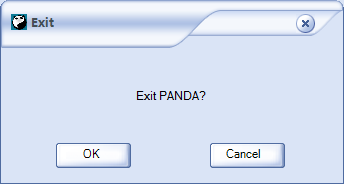


Figure 4. The exit dialog.

# Chapter 3. Main Interface

## 3.1 Parameter setting in the *Data* interface

### 3.1.1 Configuration file

There are two ways to load data and set parameters in PANDA:

1. Write a configuration file (*.config) and load it into PANDA (Fig. 5) by choosing *Load config file*. Users can click *Select file* button to choose the configuration file or just drag the configuration file into the text box next to the *Select file* button. Then the parameters and data information will be shown in the GUI (Fig. 6).
2. Set parameters and load data manually in the GUI by choosing *New config file* (Fig. 7).


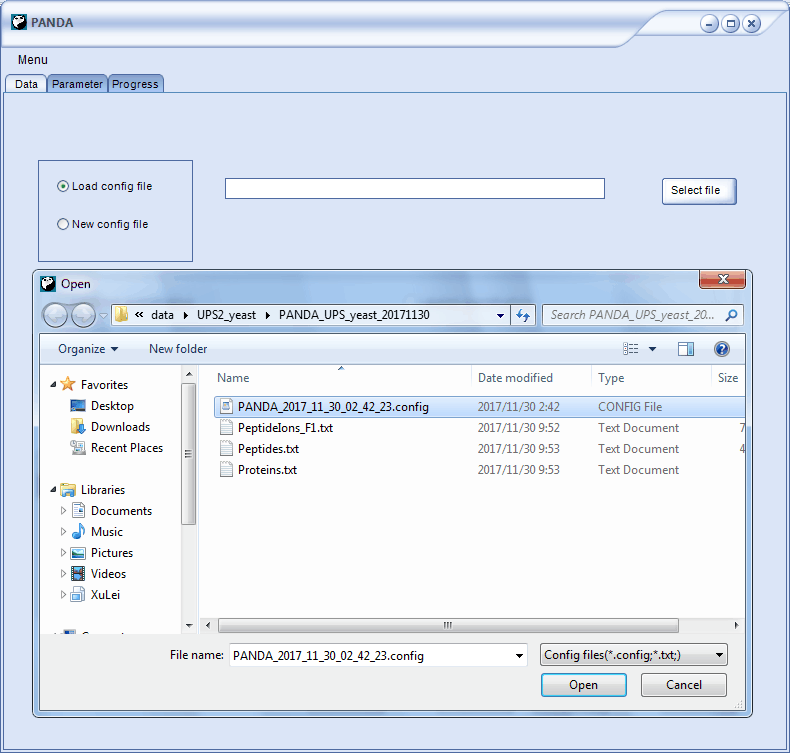


Figure 5. Load a configuration file by clicking *Select file* button.
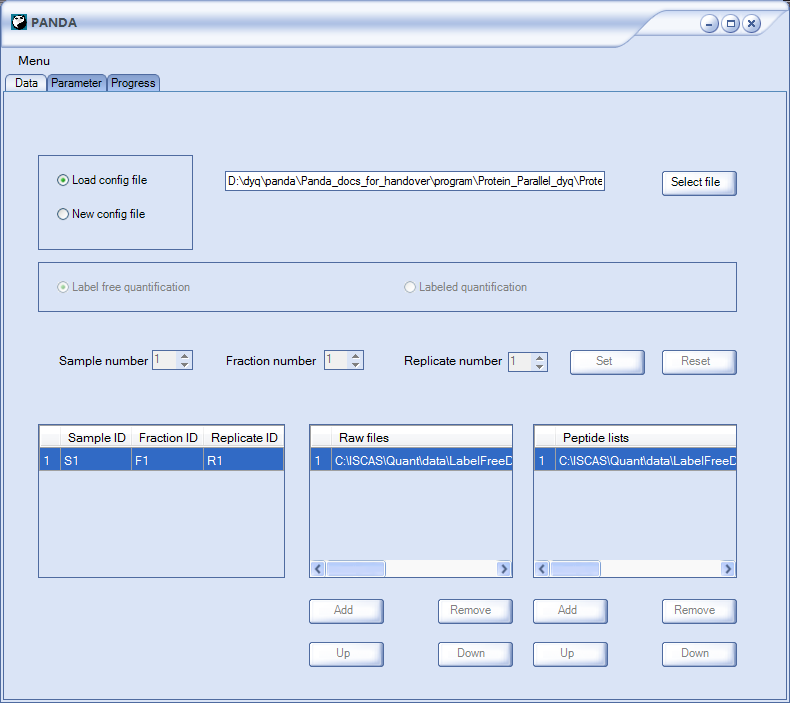


Figure 6. The parameters shown in GUI after loading a existing configuration file


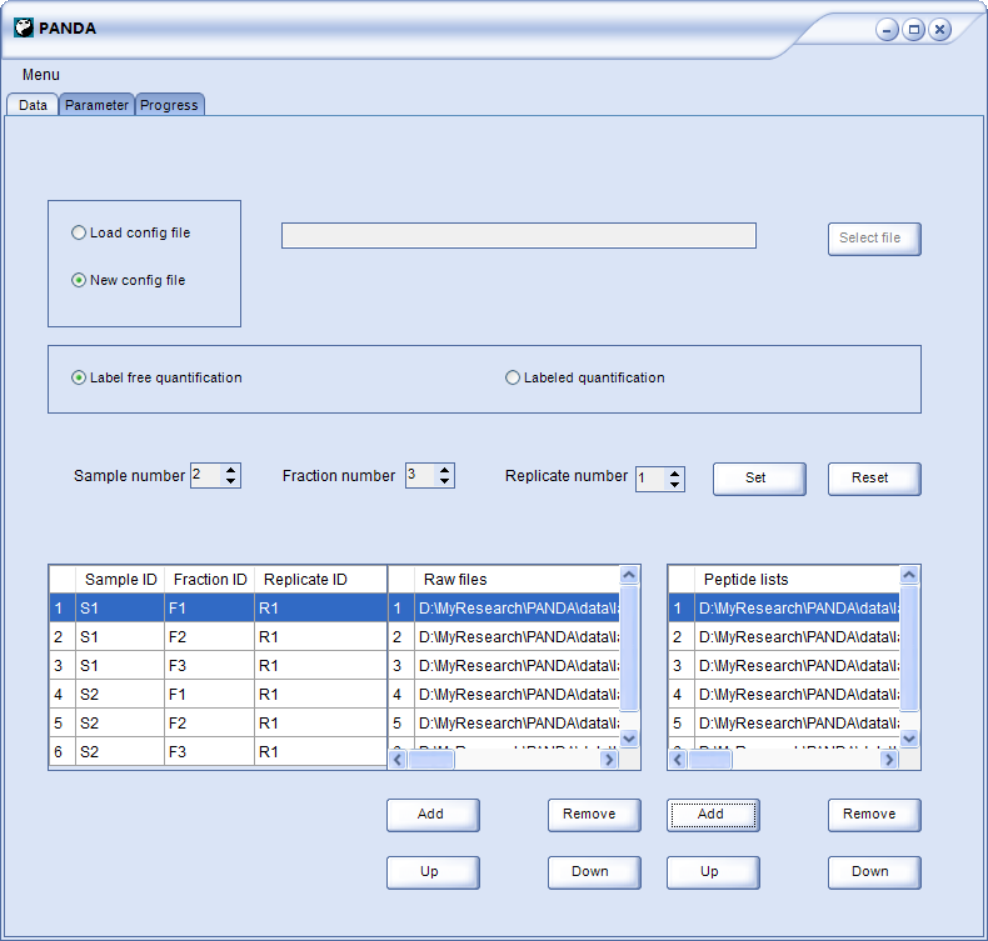


Figure 7. Setting parameters by choosing *New config file.*

### 3.1.2 Input data type

For MS data, PANDA supports Thermo raw (MSFileReader is required at first), mzXML and mzML.

For peptide identification, PANDA supports mzIdentML and the quality control results of PeptideProphet ([Keller, et al., 2002](#_ENREF_7)) and PepDistiller ([Li, et al., 2012](#_ENREF_8)). PeptideProphet can be freely obtained from Trans-Proteomic Pipeline ([Deutsch, et al., 2010](#_ENREF_5)). The original version of PepDistiller can be freely downloaded from ([Li, et al., 2012](#_ENREF_8)). We recommend users to download the new version of PepDistiller from <https://sourceforge.net/projects/pepdistiller/>.

### 3.1.3 Experiment design setting

When choosing *New config file*, users need to decide whether the experiment is labeled or not. In case of label-free quantification, users need to set the experiment design parameters, i.e. Sample number, Fraction number and Replicate number through Set button. (Fig. 8a-b). If users choose labeled quantification, Layer number, Fraction number and Replicate number parameters should be set instead (Fig. 9). Users could click *Reset* button to reset the experiment design. The detailed annotations of these parameters are shown in Table 1.


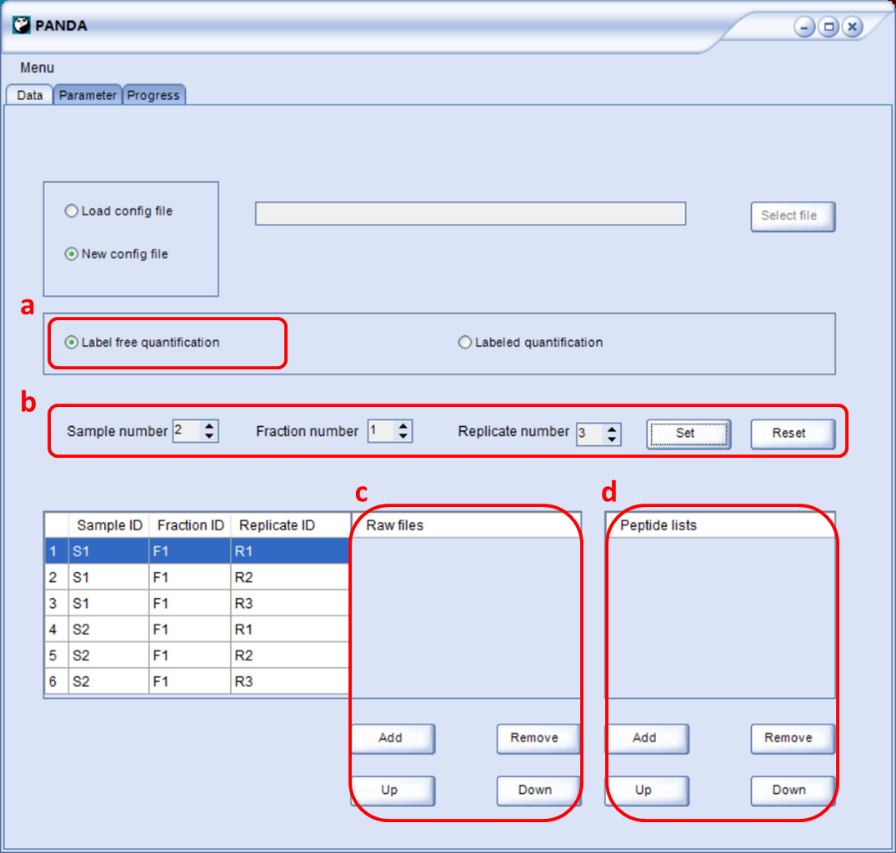


Figure 8. Experiment design setting for label-free quantification


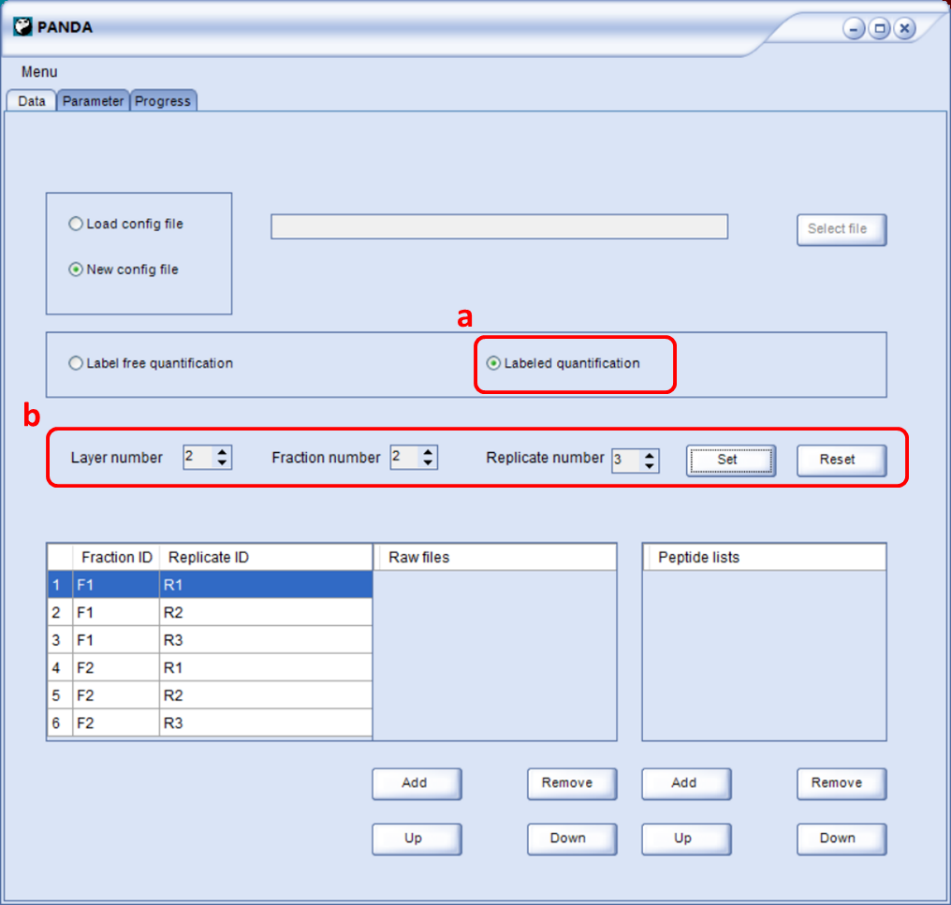


Figure 9. Experiment design setting for labeled quantification

Table 1. Annotations of the parameters about experiment design

| Parameter | Annotation |
| --- | --- |
| Sample number | Sample (Biological replicate) number |
| Fraction number | Fraction number in each sample |
| Replicate number | Technical replicate number in each fraction |
| Layer number | The labeling layer number for a specific labeling method. Default layer number is 2, representing heavy and light labels. |

### 3.1.4 Loading MS data and peptide identifications

After setting experiment design parameters, users can load or remove MS data and peptide identifications in the GUI by clicking the *Add* or *Remove* button. All the files can be reordered by clicking *Up* and *Down* buttons (Fig. 8c-d). Note, the file number of MS data and peptide identifications should be equal to the number set in experiment design, i.e. Sample number*Fraction number*Replicate number for label-free quantification or Fraction number*Replicate number for labeled quantification.

## 3.2 Parameter setting in the *Parameter* interface

### 3.2.1 Label-free quantification

Users need to fill out the parameters listed in Table 2 when choosing label-free quantification in the Data interface. Most parameters are filled in by default.

Table 2. Annotations of the parameters about peptide quantification

| Parameter | Annotation |
| --- | --- |
| XIC peak tolerance min | The peptide m/z tolerance in ppm used during XIC construction, -10 is recommended. |
| XIC peak tolerance max | The peptide m/z tolerance in ppm used during XIC construction, 10 is recommended. |
| SN cutoff | The isotope signal-to-noise ratio cutoff during XIC construction, 2 is recommended. |
| Goodness of fit cutoff | The goodness of the match between the observed and theoretical isotopic distribution, 0.6 is recommended. |
| XIC RT truncation min | The peptide retention time minimum (in minutes) during XIC construction, -5 is recommended. |
| XIC RT truncation max | The peptide retention time maximum (in minutes) during XIC construction, 5 is recommended. |
| XIC RT truncation gap | The peptide retention time gap (in minutes) during XIC construction, 0.2 is recommended. |
| Working path | The file folder path to store the results of PANDA |
| Peptide FDR | The FDR at peptide level, 0.01 is recommended. |
| Do cross quantification for label-free | A bool value to determine if cross search quantification is performed for label-free data analysis. |

### 3.2.2 Labeled quantification

For labeled quantification, users should choose the labeling method before filling in the parameters shown in Table 2. PANDA supports precursor ion labeling methods and product ion labeling methods. For precursor ion labeling, the preset methods are SILAC-K6, SILAC-K6R6, SILAC-K6R10, SILAC-K8R10, ^18^O, ^15^N, ICAT-C:^13^C (9), ICAT-D:^2^H (8), ICAT-G:^2^H (8), ICAT-H:^13^C (6), ICPL:^13^C (6), ICPL:^13^C (6) ^2^H (4), and ICPL:^2^H (4). For product ion labeling, iTRAQ 4-plex, iTRAQ 8-plex, TMT 2-plex, TMT 6-plex and TMT 10-plex are provided (Fig. 10).


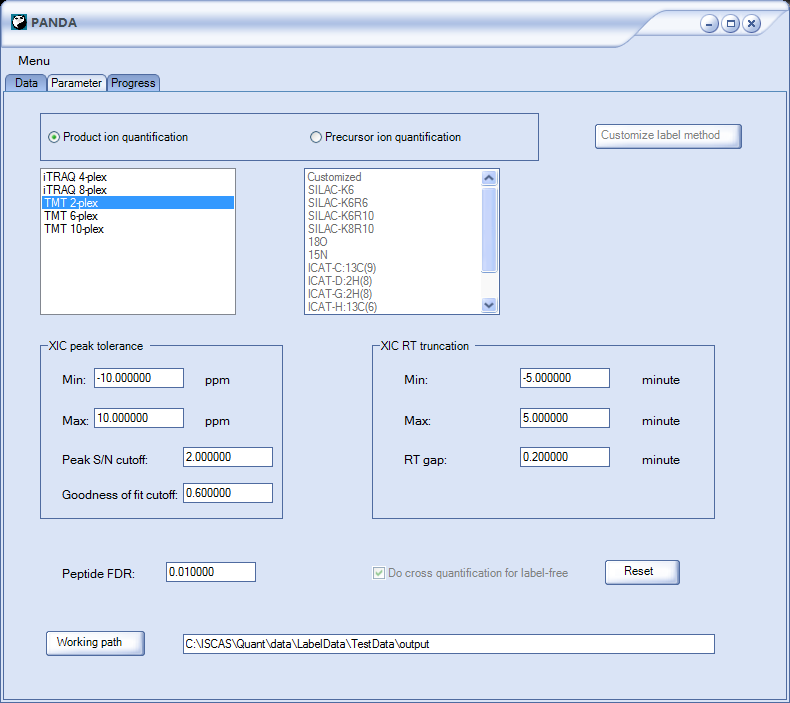


Figure 10. The *Parameter* interface.

Furthermore, users can define their own labeling methods in PANDA by choosing *Customized* item in Precursor ion quantification and clicking *Customize label method* button (Fig. 11). Then the dynamic modifications read from the peptide identification files will be shown in the dialog for users to choose (Fig. 12). And for a user-defined labeling method, the parameters including its position, modification site, modification mass and labeling layer are required to fill in manually.


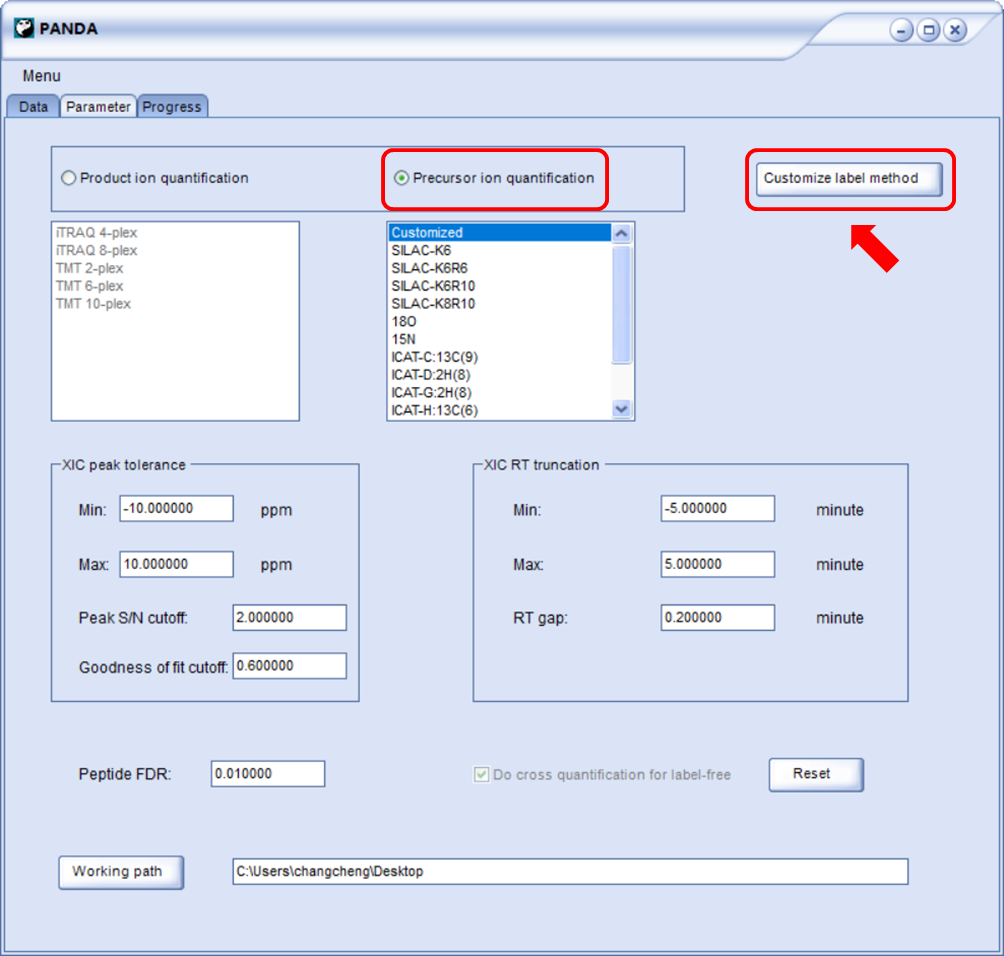


Figure 11. The procudures to customize a new labeling method.


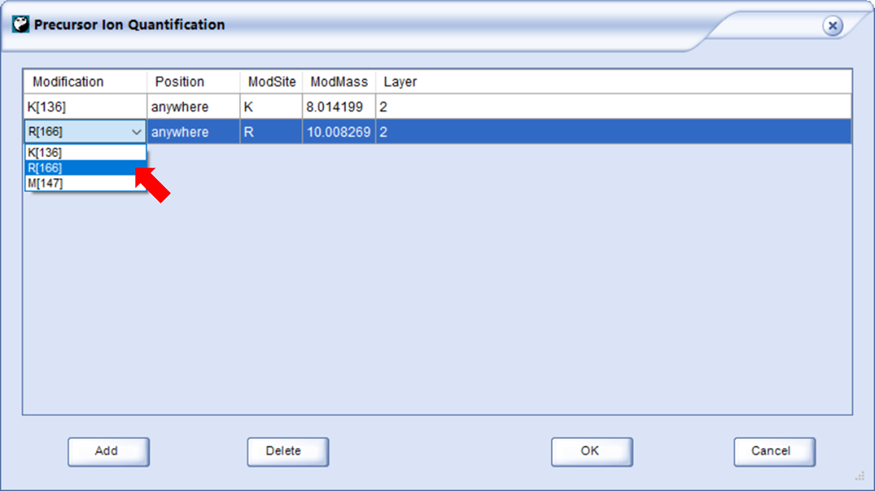


Figure 12. The dialog for user-defined labeling method.

## 3.3 Parameter setting in the *Progress* interface

In the *Progress* interface, users can set the threads used in PANDA (Fig. 13 and Fig. 14). As shown in Table 3, for label-free quantification users can set the thread number for each fraction set, each fraction and cross search quantification. And for labeled quantification, only the thread in each fraction set or fraction can be set (Fig. 14). Note: (1) The max thread to choose in the GUI is the max thread in the computer by default. (2) The max cached file number is the MS file number loaded in PANDA. Users should choose a proper value in case the program is crashed due to memory overflow. (3) In PANDA, fraction set is defined as a set of MS data containing all the samples and replicates in each fraction. For example, if the sample number is 5, fraction number is 3, replicate number is 2. Then we got three fraction sets. Each of them contains 10 (5 sample * 2 replicate) MS files.


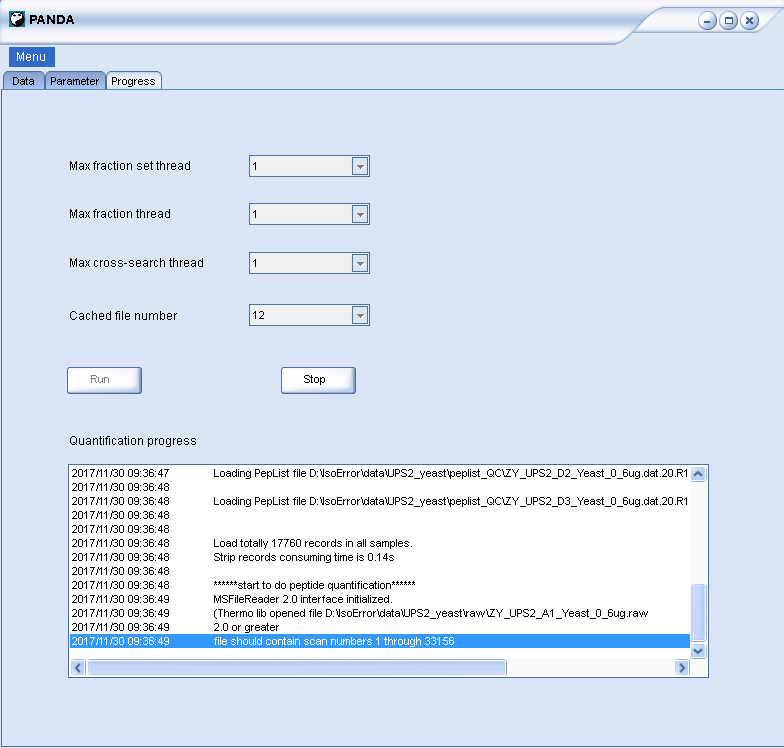


Figure 13. The *Progress* interface for label-free quantification.


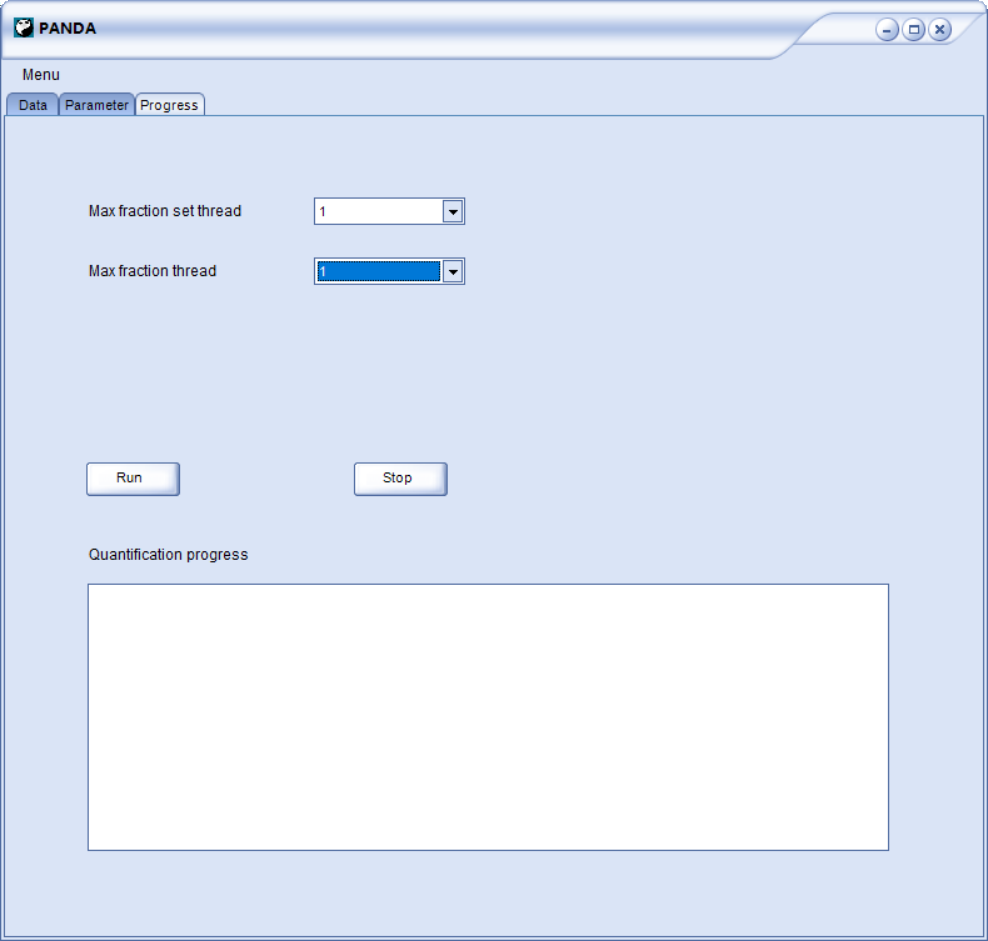


Figure 14. The *Progress* interface for labeled quantification.

Table 3. Annotations of the parameters in the *Progress* interface

| Parameter | Annotation |
| --- | --- |
| MaxFSetThread | Max thread number in each fraction set |
| MaxFractionThread | Max thread number in each fraction |
| CrossQuantMaxThread | Max thread number in cross search quantification for label-free |
| CachedSize | File number read in the cache (label-free quantification only) |

After all the parameters in the *Data*, *Parameter* and *Progress* interfaces are set, users can click *Run* button to run PANDA directly and click *Stop* button to stop PANDA by force. Once clicking *Run* button, all the parameters set in GUI will be written down as a configuration file named using the computer local time (e.g. PANDA_2017_11_30_02_42_23.config). The configuration file is saved in the same folder of PANDA program. Meanwhile, the basic information during the quantification will be shown in the *Quantification progress* text box (Fig. 13). When all the calculations are done, an alert dialog will show (Fig. 15).


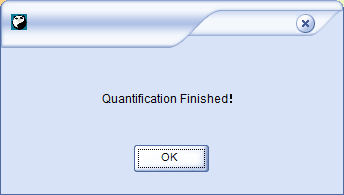


Figure 15. The dialog when quantification is done.

# Chapter 4. Output Files

Once the calculation is done, PANDA generates several files in the result directory (Table 4).

Table 4. Annotations of PANDA quantification results.

| File name | Annotations |
| --- | --- |
| Proteins.txt | The protein quantification information. |
| Petides.txt | The nonredundant peptide quantification information. |
| PeptideIons_FX.txt | The peptide ion quantification information in the X-th fraction. |
| PeptideIons_FX_XIC.xml | The XIC data information for every peptide ion quantified in the X-th fraction. The XML file is used for XIC visualization. |
| Config file | The configuration file containing all the parameters set in GUI. |

Note that the X represents the fraction index of parameter setting in the *Data* interface.

# Chapter 5. Support Services

## 5.1 Contact

For any questions involving PANDA, please contact Dr. Cheng Chang (Email: [1987ccpacer@163.com](mailto:1987ccpacer@163.com) or [1987ccpacer@gmail.com](mailto:1987ccpacer@gmail.com)).

## 5.2 Copyright

This software product is developed by Dr. Cheng Chang from the National Center for Protein Sciences (Beijing)-Bioinformatics group. All titles and intellectual property rights, which is generated by the software product including, but not limited to, relative images, data, texts, additional program and other software products (dll, exe, etc.), incidental help materials, and any copies of the Software Products are protected by Copyright Law of People’s Republic of China and international copyright treaties and other intellectual property laws and treaties. Users only get the right to use this software product for non-commercial uses.

# Chapter 6. References

Chang, C.*, et al.* (2018) PANDA-view: An easy-to-use tool for statistical analysis and visualization of quantitative proteomics data, *Bioinformatics*.

Chang, C.*, et al.* (2014) SILVER: an efficient tool for stable isotope labeling LC-MS data quantitative analysis with quality control methods, *Bioinformatics*, **30**, 586-587.

Chang, C.*, et al.* (2016) Quantitative and In-Depth Survey of the Isotopic Abundance Distribution Errors in Shotgun Proteomics, *Anal Chem*, **88**, 6844-6851.

Deutsch, E. (2008) mzML: a single, unifying data format for mass spectrometer output, *Proteomics*, **8**, 2776-2777.

Deutsch, E.W.*, et al.* (2010) A guided tour of the Trans-Proteomic Pipeline, *Proteomics*, **10**, 1150-1159.

Jones, A.R.*, et al.* (2012) The mzIdentML data standard for mass spectrometry-based proteomics results, *Mol Cell Proteomics*, **11**, M111 014381.

Keller, A.*, et al.* (2002) Empirical statistical model to estimate the accuracy of peptide identifications made by MS/MS and database search, *Anal Chem*, **74**, 5383-5392.

Li, N.*, et al.* (2012) PepDistiller: A quality control tool to improve the sensitivity and accuracy of peptide identifications in shotgun proteomics, *Proteomics*, **12**, 1720-1725.

Pedrioli, P.G.*, et al.* (2004) A common open representation of mass spectrometry data and its application to proteomics research, *Nat Biotechnol*, **22**, 1459-1466.

Wang, J.*, et al.* (2017) In-depth method assessments of differentially expressed protein detection for shotgun proteomics data with missing values, *Scientific reports*, **7**, 3367.

Zhang, W.*, et al.* (2012) LFQuant: A label-free fast quantitative analysis tool for high-resolution LC-MS/MS proteomics data, *Proteomics*, **12**, 3475-3484.
